# Detection of CCR5Δ32 Mutant Alleles in Heterogeneous Cell Mixtures Using Droplet Digital PCR

**DOI:** 10.3389/fmolb.2022.805931

**Published:** 2022-02-21

**Authors:** Alyona Sorokina, Alexander Artyuhov, Alexandra Goltsova, Erdem Dashinimaev

**Affiliations:** ^1^ Center for Precision Genome Editing and Genetic Technologies for Biomedicine, Pirogov Russian National Research Medical University, Moscow, Russia; ^2^ Lomonosov Moscow State University, Moscow, Russia; ^3^ Koltzov Institute of Developmental Biology, Russian Academy of Sciences, Moscow, Russia; ^4^ Moscow Institute of Physics and Technology (State University), Dolgoprudny, Russia

**Keywords:** CCR5, CD195, CCR5Δ32, CCR5-delta32, CRISPR/Cas9, droplet digital PCR, ddPCR

## Abstract

The C-C chemokine receptor type 5 (CCR5 or CD195) is one of the co-receptor binding sites of the human immunodeficiency virus (HIV). Transplantations of hematopoietic stem cells with the CCR5Δ32 knockout mutation could represent an effective tool for the complete cure of HIV; these methods having passed the stage of proof-of-principle. At the same time, using the modern CRISPR/Cas9 genome editing method, we can effectively reproduce the CCR5Δ32 mutation in any wild-type cells. Thus, the task of searching for and accurately quantifying the content of mutant CCR5Δ32 alleles in heterogeneous cell mixtures becomes relevant. In this study, we describe the generation of an artificial CCR5Δ32 mutation using CRISPR/Cas9 followed by multiplex droplet digital polymerase chain reaction (ddPCR) to quantify its content in cell mixtures. The system we have developed allows us to quickly and accurately measure the content of cells with the CCR5Δ32 mutation, down to 0.8%.

## Introduction

The transmembrane protein CCR5 (CD195) is one of the co-receptor binding sites of human immunodeficiency virus (HIV) (Liu et al., 1996; Venuti et al., 2017). There is a mutant form of the CCR5 gene resulting from a deletion of 32 nucleotides in the coding sequence (CCR5Δ32) that causes a frameshift, the appearance of untimely stop codons and knockout of the gene function (Hütter et al., 2011). This mutation is present in approximately 10 and 1% of the population of Northern Europe in heterozygous and homozygous variants, respectively (Stephens et al., 1998; Trecarichi et al., 2006; Muxel et al., 2008). The knockout of this gene apparently has no significant effect on the health of its carriers, but is a factor in the immunity of T cells to the human immunodeficiency virus (HIV-1), more precisely to its R5 strain (the most common and contagious one) (Balotta et al., 1997; Michael et al., 1997; Barmania and Pepper, 2013; Surdo et al., 2013; Qi et al., 2018). Because acute lymphoblastic leukemia is common among HIV-1 carriers, such patients need bone marrow replacement after chemotherapy or radiation therapy (Forghieri et al., 2020). If an immune-compatible donor is a homozygous carrier of the rare CCR5Δ32 mutation, then the virus can be completely eliminated from the HIV-1 patient. This strategy has already passed the “proof-of-principle” stage, in several similar cases in Berlin and London (Hutter et al., 2009; Jilg and Li, 2019; Gupta et al., 2020). Analysis of the grafted cell content and its expansion in the patient’s body is an important component of further monitoring of the patient’s health. At the same time, the development of genome editing technologies (ZNF, TALEN, CRISPR/Cas9) makes it possible to artificially create the CCR5Δ32 mutation in any cells from any donor, and this could provide the basis for innovative treatments for HIV-1 infections (Liu et al., 2017; Li et al., 2020). A potential treatment strategy in this case could be the creation of an artificial CCR5Δ32 mutation in autologous or immunocompatible hematopoietic stem cells or induced pluripotent stem cells (and their differentiation into HSCs) followed by transplantation. Thus, there is a need to create systems for accurate quantification of CCR5Δ32 mutant alleles in heterogeneous cell mixtures that are both efficient and simple enough to translate the method into clinical diagnostic laboratories.

Digital droplet PCR could represent a suitable method for this purpose, as it allows accurate calculation of the number of matrix copies in the original mixture and has already been applied to similar cases, when searching for oncogenic mutations (Findlay et al., 2016). Furthermore, different approaches (ddPCR; quantitative real-time PCR or qPCR) have been applied to quantitate low-levels of HIV DNA for HIV reservoir diagnostics, including for the evaluation of potential HIV cure after CCR5-Δ32/Δ32 allogeneic hematopoietic stem-cell transplantation (HSCT) during an antiviral treatment interruption (Henrich et al., 2012; Gupta et al., 2020). Previously, multiplex end-point PCR and high-performance real-time PCR have been used as convenient methods for screening HIV patients for the protective CCR5Δ32 mutation in biallelic or monoallelic variants (Nischalke et al., 2004; Rosi et al., 2015). In the present work, we aimed to develop an efficient, fast, and sufficiently accurate method for assessing the content of mutant CCR5Δ32 cells in heterogeneous cell mixtures using digital droplet PCR. For this purpose, we created an artificial CCR5Δ32 mutation in the MT4 cell line using the CRISPR/Cas9 genome editing system.

## Materials and Methods

### Cell Culture and Genomic DNA Extraction

The MT-4 human T-cell line was kindly gifted from Dr. D.N. Nosik (Gamaleya Research Institute of Epidemiology and Microbiology of the Russian Academy Sciences, Moscow, Russia). Cells were cultured in Roswell Park Memorial Institute medium (RPMI-1640; Gibco, Thermo Scientific, Waltham, Massachusetts, United States; Cat. no. 11875093), containing 10% fetal bovine serum (FBS; HyClone, Capricorn Scientific, Helicon, Moscow, Russia; Cat. no. FBS-22A) and maintained in a humidified incubator with 5% CO2 at 37°C. Genomic DNA was extracted using the phenol-chloroform method and the «ExtractDNA Blood and Cells Kit» (Evrogen, Moscow, Russia; Cat. no. BM011). The DNA concentration and purity parameters were measured with a NanoPhotometer P-Class P360 (Implen, Munich, Germany).

### Construction of CCR5-Targeting gRNAs

Relevant gRNA sequences were found from Kang et al., 2016: CCR5-7 CAG​AAT​TGA​TAC​TGA​CTG​TAT​GG and CCR5-8 AGA​TGA​CTA​TCT​TTA​ATG​TCT​GG. Such gRNA oligonucleotides were annealed and phosphorylated simultaneously using T4 polynucleotide kinase (NEB, Ipswich, Massachusetts, United States; Cat. no. M0201S) and C1000 Touch Thermal Cycler (Bio-Rad, Hercules, California, United States) for 30 min at 30°C, for 5 min at 93°C and, finally, with a decreasing ramp speed of 5°C/s from 20°C to 4°C. The pU6-gRNA vector (see plasmid map and sequence in the Supplementary Figure S1, Supplementary File S1) was linearized by BsmBI digestion (NEB, Ipswich, United States; Cat. no. R0580) and ligated with the annealed oligonucleotides into the backbone using T7 DNA ligase (NEB, Ipswich, United States; Cat. no. M0318S) in a C1000 Touch Thermal Cycler (Bio-Rad, Hercules, United States) according to the protocol: for 10 min at 16°C and for 1 min at 10°C over three cycles. The ligated DNA was purified using the «Isolate II PCR and Gel Kit» (Bioline or Meridian Bioscience, Cincinnati, Ohio, United States; Cat. no. BIO-52059) and then used for the chemical transformation of *Escherichia coli* XL1-Blue cells (Evrogen, Moscow, Russia; Cat. no. CC001) following the standard manufacturer’s instructions. Plasmids with successfully inserted gRNAs were obtained after extraction using a «Plasmid Midiprep 2.0 Kit» (Evrogen, Moscow, Russia; Cat. no. BC124) and verification using Sanger sequencing (Evrogen, Moscow, Russia).

### Cas9-gRNA Electroporation Procedure

10 µg of pCas9-IRES2-EGFP (see plasmid map and sequence in the Supplementary Figure S2, Supplementary File S2) were mixed with 5 µg of pU6-gRNA-CCR5-7 and 5 µg of pU6-gRNA-CCR5-8 in the presence of «Gene Pulser Electroporation Buffer» (Bio-Rad, Hercules, United States; Cat. no. 1652677) or Dulbecco’s Phosphate-Buffered Saline (DPBS; PanEco, Moscow, Russia; Cat. no. Р060p) followed by transfer to 0.4 cm «Gene Pulser MicroPulser Electroporation Cuvettes» (Bio-Rad, Hercules, United States). After this, 6 × 10^6^ MT4 cells were added to the Cas9-gRNA mix and the whole mixture was electroporated using a Gene Pulser Xcell (Bio-Rad, Hercules, United States) with the following settings: 275 V, 5 ms, three pulses. Upon electroporation, the cells were incubated in RPMI-1640 (Gibco, Thermo Scientific, Waltham, United States; Cat. no. 11875093) complemented with 10% FBS (Capricorn Scientific, Helicon, Moscow, Russia; Cat. no. FBS-22A) for 48 h before cell sorting.

### MT4 Cell Sorting and Cloning

The transfected MT4 cell suspension was collected and separated using fluorescence-activated cell sorting (FACS) in an S3 Cell Sorter (Bio-Rad, Hercules, United States). The EGFP labeled cell population was cloned manually using a multichannel pipette to dispense cells into 96-well plates by limiting dilution in order to generate a monoclonal cell lines as most wells contain only a single cell. After that, the 96-well plates were incubated for 14 days under standard conditions. During this period, we carefully visually screened the wells for two or more cells in the well of 96-well plate. This is quite easy to do because MT-4 cells have the ability to form spheres from the derivatives of a single cell divide. Accordingly, when 2 cells came into the one well, we saw two spheres, but this was also a rather rare event. In the case of finding such wells, we excluded such variants from further study.

### Screening of CCR5-Δ32 Alleles From MT4 Clones

After obtaining monoclonal cell lines, we amplified them and isolated DNA, after which we amplified the studied fragment of the CCR5 locus by PCR using following primers: forward CCC​AGG​AAT​CAT​CTT​TAC​CA and reverse GAC​ACC​GAA​GCA​GAG​TTT. For further sequencing, we applied TA-cloning, since this approach allows for better sequencing efficiency and allows us to see potential contamination of monoclonal lines with other cell types (e.g. homozygous mutants with wild-type cells).The CCR5 amplified DNAs were purified using the «Isolate II PCR and Gel Kit» (Bioline, Memphis, United States; Cat. no BIO-52059). Amplicons were inserted into the pAL2-T vector (Evrogen, Moscow, Russia; Cat. no. TA002) through the T4 DNA ligase (NEB, Ipswich, United States; Cat. no. M0202L) reaction according to the manufacturer’s protocol. The obtained DNA ligation products were used for the chemical transformation of *Escherichia coli* XL1-Blue cells (Evrogen, Moscow, Russia; Cat. no. CC001) as per standard methodology. A suspension of the transformed cells was transferred on the surface of LB-agar plates (solid Luria-Bertani medium; all ingredients were acquired from Helicon, Moscow, Russia) with selective antibiotic (ampicillin, Cat.no: A8351, Sigma), IPTG (Cat.no: I1284, Sigma) and X-Gal (Cat.no: 203782, Sigma) according to the blue-white screening procedure. The Petri dishes were kept in a dry-air thermostat for 14–16 h at 37°C. For subsequent PCR analysis with M13 primer, 6–8 white colonies were selected. An overnight culture of bacteria containing the target site was grown in LB medium (liquid Luria-Bertani medium; all ingredients were acquired from Helicon, Moscow, Russia) plus selective antibiotic at 37°C and 250 rpm. Plasmids were prepared using the «Isolate II Plasmid Mini Kit» (Bioline, Memphis, United States; Cat. no BIO-52056) and sequenced by Sanger’s approach (Evrogen, Moscow, Russia). For each clone studied, we sequenced at least 10 independently obtained TA plasmid samples. The data were submitted to SnapGene Viewer version 4.1.9 software (from Insightful Science, San Diego, California, United States; available at snapgene.com) and analyzed.

### CCR5 Gene Copy Number Measurement (CNV Assay)

The ddPCR reactions were performed in a 22 μl reaction mixture containing 2X droplet digital PCR supermix (no dUTP; Bio-Rad, Hercules, California, United States; Cat. no. 186-3024), 250 nmol of each probe labeled with R6G or FAM, 450 nmol primers, and 5 μl containing 25–80 ng of gDNA. Nuclease-free water (Evrogen, Moscow, Russia; Cat. no. PB207M) was added to achieve the final volume. Droplets were generated using the «QX200™ AutoDG Droplet Digital PCR System» (Bio-Rad, Hercules, California, United States). Amplifications were performed with the following cycling conditions: one cycle at 95°C (2°C/s ramp) for 10 min, 40 cycles at 94°C (2°C/s ramp) for 30 s with the annealing step at either 56°C for 90 s or at 58°C for 60 s, followed by one cycle at 98°C (2°C/s ramp) for 10 min. All samples were analyzed in duplicate.

### Droplet Digital PCR

The PCR reactions were performed in a 22 μl reaction mixture containing 2X droplet digital PCR supermix (no dUTP; Bio-Rad, Hercules, California, United States; Cat. no. 186-3024), 250 nmol of each probe labeled with R6G or FAM, 900 nmol primers, and 5 μl containing 25–80 ng of gDNA. Nuclease-free water (Evrogen, Moscow, Russia; Cat. no. PB207M) was added to achieve the final volume. Samples were analyzed for CCR5Δ32 mutations in exon 3. Droplets were generated using the «QX200 ™ AutoDG Droplet Digital PCR System» (Bio-Rad, Hercules, United States). Amplifications were performed with the following cycling conditions: one cycle at 95°C (2°C/s ramp) for 10 min, 40 cycles at 94°C (2°C/s ramp) for 30 s with the annealing step at either 56°C for 90 s or at 58°C for 60 s, followed by one cycle at 98°C (2°C/s ramp) for 10 min. All samples were analyzed in duplicate.

### Primers and Probes

The M13 primer (FW GTT​GTA​AAA​CGA​CGG​CCA​GTG and RV AGC​GGA​TAA​CAA​TTT​CAC​ACA​GGA) was obtained from the standard primer collection for DNA sequencing (Evrogen, Moscow, Russia). Genomic DNA from CRISPR/Cas9 treatment cells was used for the CCR5 gene amplification with the following primers: forward CCC​AGG​AAT​CAT​CTT​TAC​CA and reverse GAC​ACC​GAA​GCA​GAG​TTT. The primer sequences for the ddPCR were the following: CCR5-NLS-FW GCGTCTCTCCCAGGA and CCR5-NK-RV1 CAA​CCT​GTT​AGA​GCT​ACT​G. The CCR5-specific primers were adopted to distinguish between the CCR5 target locus and a highly similar site in CCR2 when local similarity searching in BLAST (Basic Local Alignment Search Tool; NCBI, Bethesda, Maryland, United States) was performed. The annealing temperature was determined using gradient PCR. The fluorescent probes CCR5-mut (FAM)- CAGTCAGTATCAATTCTGGAAGA-(BHQ1) and CCR5-wt (R6G)-CTGGGCTCCCTACAACAT- (BHQ2) were synthesized using «DNA-Syntez» (Moscow, Russia). All primers were designed using Primer-BLAST (Ye et al., 2012; NCBI, Bethesda, Maryland, United States) and synthesized by «Evrogen» (Moscow, Russia). For CNV assay following primers and probes for APP were used: forward TTG​GTT​GTC​CTG​CAT​ACT​TT and reverse AGC​ACA​GGA​TGA​ACC​AGA, (FAM)-CTCTGAAGTGAAGATGGATGC-(BHQ1), (R6G)- CCACTACTGTTTGTCTTGCC-(BHQ2).

### ddPCR Dilution Series

To evaluate the sensitivity of the ddPCR assay, the MT4 cells derived from clones presumed to contain the homozygous CCR5Δ32 mutation were chosen and mixed with wild-type MT4 cells and then diluted as follows: 50, 10, 2%, 0.4% and 0.08% (mutation-type in wild-type and vice versa) followed by gDNA extraction. In addition, DNA solutions isolated from the wild-type MT4 cell line were serially diluted with DNA from MT4 cells with CCR5Δ32, in six dilution steps starting at 20% with a dilution factor of 5 (20, 4, 0.8, 0.16, 0.032, and 0.0064%) and vice versa. A total of three negative controls (wild-type only and mutant only and NTC) were routinely included to reduce false positive results.

### Statistical Analysis

The DNA serial dilution experiments were performed in the format of two independent biological repeats for each point, and also the MT4 cell dilution experiment was staged in two independent biological repeats for each point. For the ddPCR procedure itself, we staged two technical repeats for each point. ddPCR absolute quantifications of the mutant and wild-type alleles were estimated by the Poisson distribution law using QuantaSoft Analysis Software (Bio-Rad, Hercules, California, United States; Cat. no. 186-3024). Thresholds were defined based on the signal from the negative control (wild-type only and mutant only) wells and empty droplets, as described in the Droplet Digital Application Guide (Bio-Rad, Hercules, California, United States; Cat. no. 186-3024). The differences in the rates of mutant and wild-type alleles as measured by ddPCR were compared using GraphPad Prism version 9.1.2 (GraphPad Software, San Diego, California, United States) and Rstudio (Boston, Massachusetts, United States).

## Results

### Generation of CCR5Δ32 Mutation Using the CRSIPR/Cas9 System

**FIGURE 1:**
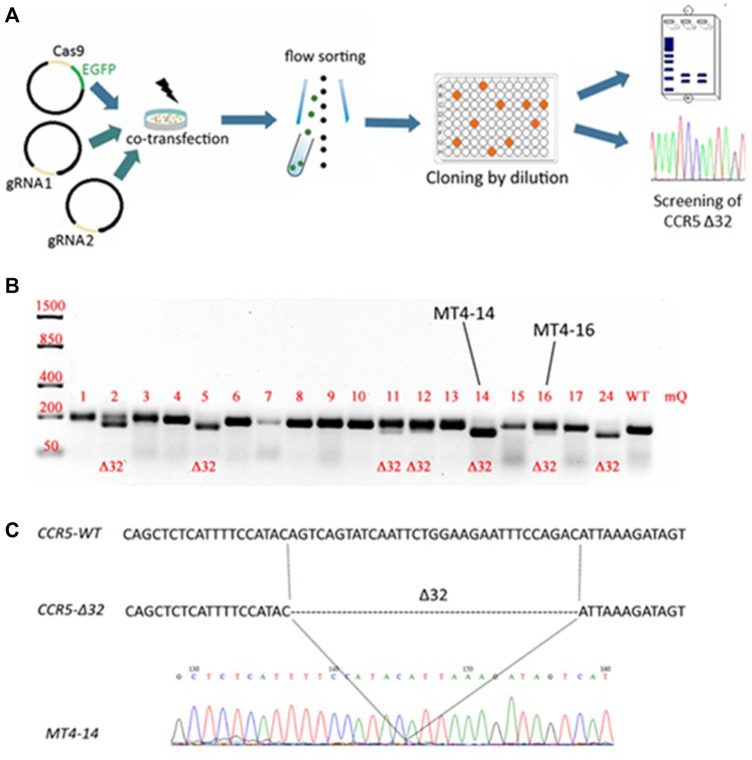
Generation of an MT4 cell line with an artificial CCR5Δ32 mutation using the CRISPR/Cas9 system **(A)** Experimental design **(B)** Analysis of the CCR5 locus containing the Δ32 mutation in the obtained clones. The CRISPR targeted CCR5 locus was PCR amplified from MT4 genomic DNA and PCR products were size-separated by electrophoresis on a 2% agarose gel. The expected amplicon size is 191-bp for WT samples, and 159-bp for CCR5Δ32 samples **(C)** Sanger sequencing of PCR amplicon of MT4-14 clone containing the 32 bp deletion in the CCR5 locus.

The CCR5Δ32 mutation in MT4 cells was created using the CRISPR/Cas9 genome editing system. For this purpose, two guide RNAs were selected to carry out two double-strand breaks flanking a 32-letter deletion in exon three of the protein-coding sequence of the CCR5 gene. The use of these guide RNAs had previously been published in an article by Kang et al. (2016). The MT4 cells were electroporated simultaneously with three plasmids, two of which (pgRNA1 and pgRNA2) expressed guide RNAs #1 and #2, respectively, and the third plasmid expressed the spCas9 protein and the green fluorescent marker protein EGFP. Transfected cells were selected by cell sorting for the green fluorescent signal and further cloned by limiting dilution (Figure 1A). The resulting clones were screened for the presence of the CCR5Δ32 mutation, and several clones with putative homozygous (e.g., MT4-5, MT4-14, MT4-24) and heterozygous (e.g. MT4-2, MT4-3, MT4-11, MT4-16) mutations were found (Figure 1B). The selected clonal lines with the CCR5Δ32 mutation was analyzed by Sanger sequencing (e.g. clonal line MT4-14, Figure 1C). In further experiments, we used this MT4-14 line as our line with a homozygous CCR5Δ32 mutation (mut^+^ or mutant MT4 cells) in addition to the original MT4 line without the mutation (wild-type or WT cells). We chose this clonal line MT4-14 to use for further development of the detection method because it showed a distinct mutant amplicon in the screening and also confirmed the homozygous state of the mutation. Because CRISPR manipulation often results in large deletions at the target loci, we could have encountered a situation where, in a putative homozygous mutant clone of MT4-14, one CCR5 allele received the desired 32-nucleotide deletion, while the other allele was so severely disrupted that it would not be detectable by PCR. For this purpose, we measured the gene copy number variation (CNV) by ddPCR and found that both mutant alleles were present in the MT4-14 line (Supplementary Figure S3.). Also, as one of the controls, we selected a line with a putative heterozygous mutation, MT4-16. To make sure that it was a heterozygous mutation and not an equal mixture of wild-type cells with homozygous mutant cells, we performed one more round of cloning with limiting dilution and made sure that the same heterozygous mutation was observed in all-new 12 clones (Supplementary Figure S4).

### Detection and Quantification of CCR5Δ32 Mutant Alleles in Cell Mixtures Using ddPCR

In order to develop a system for detecting low-presence allele variants of the CCR5 gene using digital droplet PCR, we set up a series of experiments on serial dilution of the MT4-14 cells carrying mutant alleles of the CCR5 gene. In doing so, we were therefore performing dilutions of mutant cells in “wild-type” cultures and vice versa.

**FIGURE 2:**
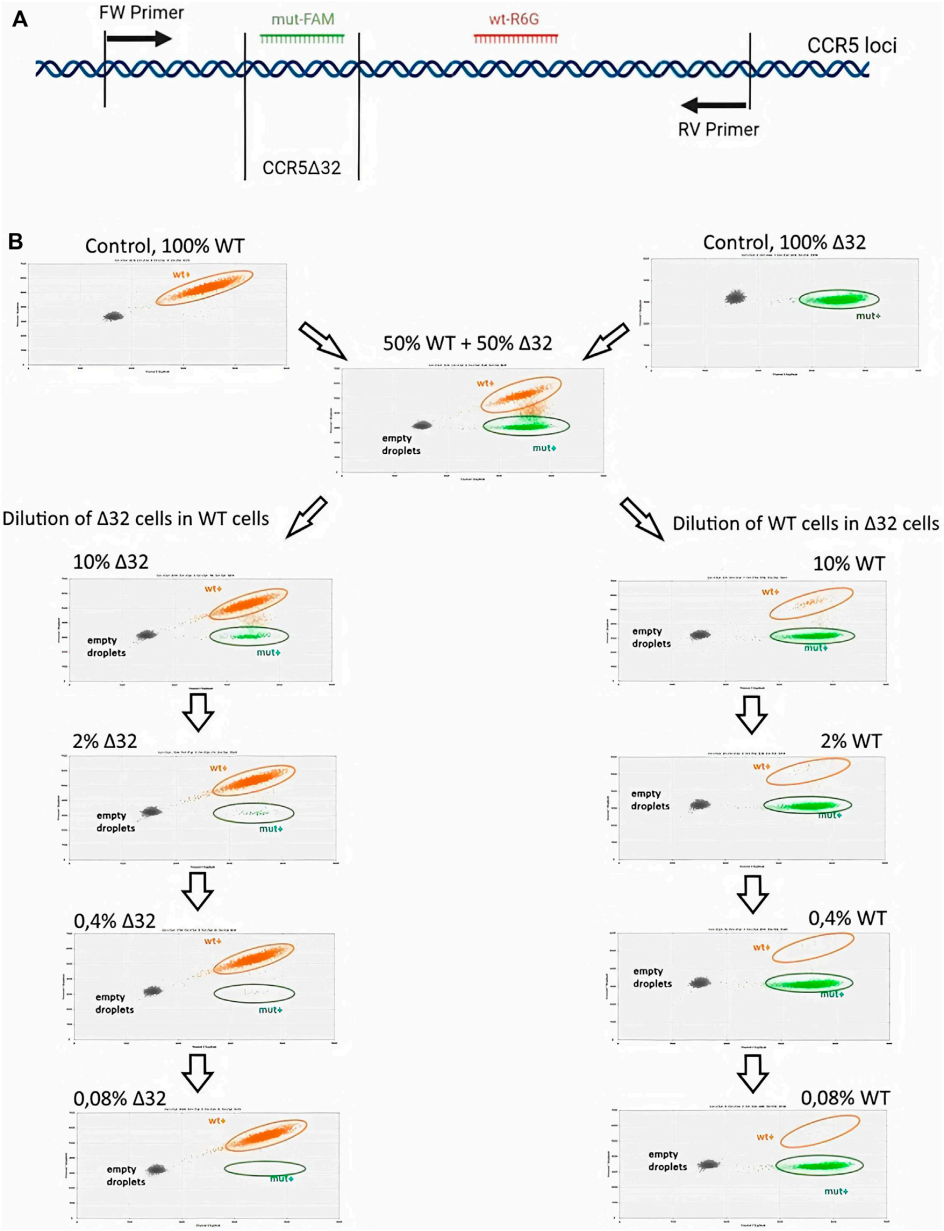
Sensitivity testing of the ddPCR assay for CCR5 alleles detection in MT4 cell mixtures after serial dilution **(A)** Schematic representation of the primers and FAM or R6G labeled probe hybridization arrangements **(B)** 2-D visualization of ddPCR results of MT4-14 cell line dilution, Δ32 cells in wild-type cells (the left-hand column) and wild-type cells in Δ32 cells (the right-hand column).

The schematic primer and probe configuration is given in Figure 2A, where amplicons with 32-bp CCR5 gene deletion in the exon three hybridized to the R6G-labelled probe, while wild-type (WT) PCR-products became marked with the FAM^+^/R6G^+^ signal. Consequently, three clusters were identified as single-positive (R6G^+^; green), double-positive (FAM^+^/R6G^+^; orange) and double-negative (grey). Additionally, in the performed ddPCR reactions, a second cluster of double positive droplets above the R6G^+^ cloud was detected as “rain”. The “rain” partially was included into the double positive droplets (Figure 2B), although the rain partitions made up only a small fraction of the total droplet number it did not make sense to exclude the rain completely from analysis. To test the validity of the assay, we performed the analysis on the putative heterozygous MT4-16 line to demonstrate the expected 50% mutant allele content. The results were generally within the required 50%, taking into account the variation obtained in biological and technical replicates (Supplementary Figure S5). We also measured the presence of false-positive signals using ddPCR analysis of NTC (no template control) variants. It is required for general monitoring for external nucleic acid contamination. The fluorescence threshold between positive and negative drops is determined by the Quantasoft software using a combined analysis of all experimental wells. Finally, we found that a very small proportion of single events (one to three drops per 15–18 thousand drops) can slip above the threshold, giving a statistically insignificant percentage of false-positives (Supplementary Figure S6).

**FIGURE 3:**
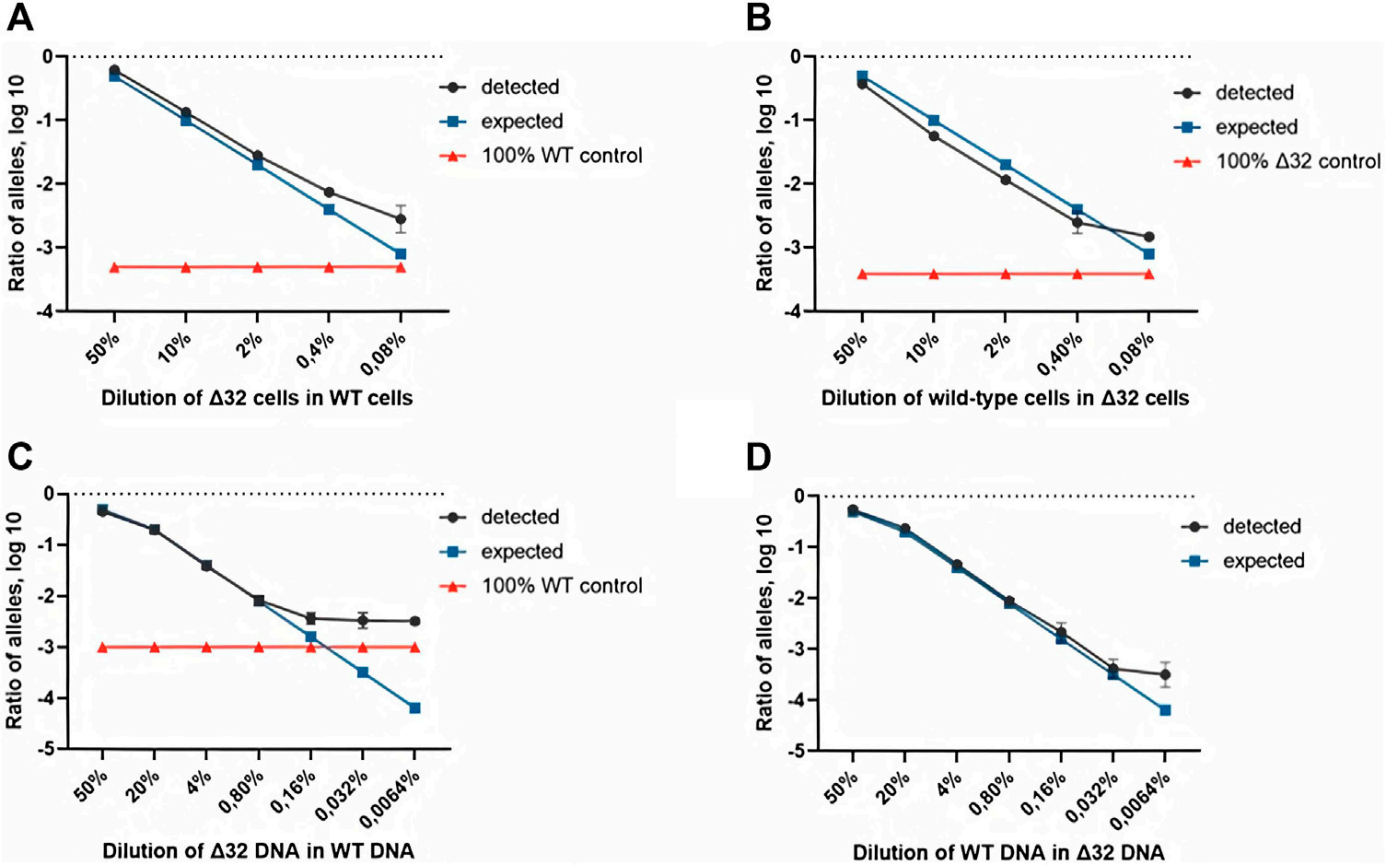
Comparison of both ddPCR assays using the MT4 cells **(A,B)** and genomic DNA **(C,D)** as test materials for serial dilution. The blue line indicates the expected ratio.

Measurements of the content of different CCR5 alleles (mutant and wild-type) using ddPCR, showed a significant decrease in the number of corresponding droplets in different dilution lines. When Δ32 cells were diluted in wild-type cells, the number of FAM-negative and R6G-positive mutant droplets decreased from 4,591 to 752, 167, 44, and 18, respectively, after dilution from 50 to 0.08% with a dilution factor of 5 (Figure 2B, left column). When wild-type cells were diluted in Δ32 cells, the number of double-positive FAM+/R6G+ droplets decreased from 3650 to 298, 62, 19, and 13, respectively, from 50 to 0.08% with a dilution factor of 5 (Figure 2B, right column). Considering the statistics (two independent biological replicates and two technical replicates for each point), as well as normalization to the total amount, we obtained data indicating a significant level of detection of the different dilution steps (Table 1; Figure 3A). Thus, we confirmed the proof-of-principle of the method for detecting CCR5Δ32 mutant cells in heterogeneous cell mixes using droplet digital PCR.

**TABLE 1:** Detection of the ratios of CCR5Δ32 mutant and wild-type alleles by ddPCR using heterogeneous cell mixture dilution.

Dilution, %	% Of wt alleles	% Of Δ32 alleles	Standard deviation
50	37.50	62.50	0,0113
Δ32 in wt			
10	86.55	13.45	0,0007
2	97.20	2.80	0,0014
0.4	99.25	0.75	0,0007
0.08	99.70	0.30	0,0014
wt in Δ32			
10	5.65	94.35	0,0035
2	1.16	98.84	0,0011
0.4	0.26	99.74	0,0010
0.08	0.15	99.85	0,0001
control, 100%			
Δ32	0.04	99.96	0,0002
wt	99.95	0.05	0,0007

Next, we conducted a series of experiments to determine the accuracy, efficiency, and detection limits of the proposed method. The typical values found in the literature range from 0.1 to 0.005% (Reid et al., 2015; Wang et al., 2015; McEvoy et al., 2018). For this purpose, we performed a series of dilutions of DNA isolated from cells with the homozygous CCR5Δ32 mutation and wild-type cells. Two independent series of dilutions were also performed, the first one was a six-step dilution of DNA from Δ32 cells into DNA from wild type cells, starting at 20% with a dilution factor of 5 (20, 4, 0.8, 0.16, 0.032, and 0.0064%), and the second one was a symmetric dilution of DNA from wild type cells into DNA from Δ32 cells, also in six steps with a dilution factor of 5 (20, 4, 0.8, 0.16, 0.032, and 0.0064%). In this part of the work, we performed measurements in four independent biological replicates and two technical replicates for each point. The ddPCR results confirmed the ability of the developed method to accurately track the decrease in the concentration of detectable CCR5 gene alleles (Supplementary Figure S7, Table 2 and Figure 3). When analyzing the obtained ddPCR results, we encountered an interesting phenomenon of unequal accuracy and search limits for different alleles. When searching for rare wild-type alleles among delta32 alleles, the accuracy according to our calculations was 0.1%, with the search limit = 0.16% (separation with control samples with *p*-value = 0.029, calculated using the Mann-Whitney criteria in RStudio). When searching for rare delta32 alleles in WT alleles, we calculated accuracy to be 0.5% with a search limit of 0.8% (separation with control samples *p*-value = 0.025, calculated using Mann-Whitney criteria in RStudio). Thus, we define the common accuracy of the developed method as 0.5% and the detection limit as 0.8%.

**TABLE 2:** Detection of the ratios of CCR5Δ32 mutant and wild-type alleles by ddPCR using genomic DNA dilution.

Dilution, %	% Of wt alleles	% Of Δ32 alleles	Standard deviation
50	55.00	45.00	0,0066
Δ32 in wt			
20	80.55	19.45	0,0060
4	96.20	3.80	0,0016
0.8	99.15	0.85	0,0017
0.16	99.63	0.37	0,0010
0.032	99.65	0.35	0,0010
0,0064	99.68	0.32	0,0005
wt in Δ32			
20	23.59	76.41	0,0090
4	4.61	95.39	0,0028
0.8	0.90	99.10	0,0012
0.16	0.23	99.77	0,0008
0.032	0.03	99.97	0,0003
0,0064	0.02	99.98	0,0002
Control			
wt	99.75	0.25	0.001
delta 32	0.00	100.00	0,0000

## Discussion

Efficient and quantitative screening of artificially modified cells is essential for genome editing workflows. At the moment, several studies have been published in which CCR5Δ32 mutations have been detected, mainly by using PCR with endpoint analysis (amplicon length measurement) (Rosi et al., 2015) and multiplex real-time PCR (using a DNA probe) (Nischalke et al., 2004). However, it is obvious that these methods cannot provide a high accuracy of quantitative detection of mutant alleles in the heterogeneous cell mixtures that may arise as a result of genome editing operations and further cell transplants. Possible competitors to the use of ddPCR are the NGS sequencing methods (Malicherova et al., 2018; Dong et al., 2018), which theoretically, with their great depth of reading, can provide the highest efficiency and accuracy. A number of studies have used NGS sequencing data for off-target and on-target editing at CCR5 locus to evaluate the results of inactivation of the CCR5 gene (Liu et al., 2017; Schwarze et al., 2021). But the disadvantage of these methods is still the high cost of analysis and the need to use bioinformatics methods when analyzing the results. This method is really necessary when figuring out the spectrum of unknown arising mutations after CRISPR/Cas9 intervention in the genome, but if we are talking about one particular known mutation (for example CCR5Δ32), then the use of NGS sequencing may be too redundant.

Mutation detection by droplet digital PCR assay has several benefits such as its cost-effectiveness and fast result turnaround time (McEvoy et al., 2018). Moreover, it requires low concentrations of sampled genomic DNA and can be used both for bulk cell populations and for the screening of single cell-derived clones (Findlay et al., 2016). However, the obvious physical limitation of this method is the number of generated droplets in one sample (about 10–18 thousand), in which the PCR reactions then take place. Taking into account the need for a negative reference cluster, as well as the peculiarities of the mathematical apparatus offered by the device manufacturer, we estimate the theoretical limit of the efficiency of mutant allele detection to be 0.05%.Although the resolution limit achieved in this study does not reach the theoretically possible limit, due to practical limitations, we believe that the satisfactory search dynamics in different dilutions with rather low search limits (0.8 and 0.16%) indicate that our selection of primer sequences and fluorescent probes was correct.

There are at least two works using ddPCR to evaluate the results of CRISPR/Cas9 systems, such as HDR/NHEJ equilibration or the occurrence of large deletions, insertions or inversions (Miyaoka et al., 2016; Watry et al., 2020). It is worth noting that both teams argue that measurement results are highly dependent on the specific experiment-the locus, the context, and the systems used. And in our study, we simply focused on one specific task: optimizing the ddPCR method to accurately count alleles with one specific mutation. Moreover, our method allows us to analyze both cells with the natural CCR5Δ32 mutation already used in clinics and cells with an artificially created mutation using CRISPR/Cas9 systems. In our opinion, given the great clinical importance of this mutation for the treatment of such a socially significant disease as HIV infection, our findings will be of interest to both clinical and scientific researchers. Thus, we believe that our work has a certain degree of novelty and could potentially find useful applications in biotechnology.

In general, the ddPCR reaction, as designed, generated two amplicons from one primer set by using the fluorescent signal from a probe specific for each amplicon. Well-designed assays exclude additional clouds between the double positives and single positive droplets visible on the 2D plots, so the presence of this cluster indicated sample-related problems. For example, such clouds may occur as part of mismatched hybridization of the probes. On the other hand, the loss of probe specificity could be explained by optical bleed-through or differences in the initial steps involving variation in the sample concentration, in which case the likelihood of copies of each amplicon being randomly distributed into each partition increases as the concentrations rise. This will lead to a tilting or lifting of the single-positive or double-positive partitions on the 2D plot (Whale et al., 2016).

Also, it should be noted that the limitations of the developed detection method include its specificity exactly to CCR5Δ32 mutations, given the binding sites of primers and probes. At the same time, many other CRISPR-mediated CCR5 knockout strategies have been described (Kang et al., 2015; Yu et al., 2018; Xiao et al., 2019; Liu et al., 2020; Lin et al., 2021) to which our detection system would not be specifically applicable, in contrast to the more generalized NGS method of sequencing the entire CCR5 locus. However, we consider that the application of alternative CCR5 knockout strategies is still very far from clinical practice, as there is a very long way to prove the biosafety of such mutant cells. The use of cells with the CCR5Δ32 mutation is already in clinical application (Gupta et al., 2020), and we believe that researchers and clinical workers can exploit our system already now. At least two other mutations in CCR5 are also known to knockout the gene and provide immunity to HIV-1 (Quillent et al., 1998), but the prevalence of carriers of these mutations is extremely low to consider them as a real resource for transplantations. Various other mutations in CCR5 are described in articles, studying genetic polymorphisms in the open reading frame of the CCR5 in populations all over the world (Kageyama et al., 2001; Zheng et al., 2002; Ronsard et al., 2019). Certain mutations are shown to slow down the HIV progression. Nevertheless, these mutations and their influence on HIV progression require additional investigation.

## Conclusion

In this manuscript, we have reported on implementing ddPCR assays based on the serial dilution of MT4 cells and genomic DNA for CCR5Δ32 mutation detection. In our approach, we designed probes labeled with two different fluorescent dyes (FAM or R6G) resulting in optimal separation of the clusters on the 2D plots. As a result of this research, we have demonstrated the efficacy of droplet digital PCR assays for the sensitive detection of CCR5Δ32 mutations using genomic DNA samples or heterogeneous cell mixtures by CRISPR/Cas9 promoted experiments with the MT4 cell line. Thus, we have developed an efficient, rapid, and accurate method for detecting mutant CCR5Δ32 cells in heterogeneous cell mixtures, which can be used in clinical bone marrow transplantation methods for treating HIV-1 infections to control donor cell repopulation, or in laboratory genome editing efforts to evaluate the effectiveness of the method.

## Data Availability

The original contributions presented in the study are included in the article/Supplementary Material, further inquiries can be directed to the corresponding author.

## References

[B1] Balotta C. Bagnarelli P. Violin M. Ridolfo A. L. Zhou D. Berlusconi A. (1997). Homozygous Δ32 Deletion of the CCR-5 Chemokine Receptor Gene in an HIV-1-Infected Patient. AIDS 11 (10), F67–F71. 10.1097/00002030-199710000-00001 9256936

[B2] Barmania F. Pepper M. S. (2013). C-C Chemokine Receptor Type Five (CCR5): An Emerging Target for the Control of HIV Infection. Appl. Translational Genomics 2, 3–16. 10.1016/j.atg.2013.05.004 PMC5133339 27942440

[B3] Dong L. Wang S. Fu B. Wang J. (2018). Evaluation of Droplet Digital PCR and Next Generation Sequencing for Characterizing DNA Reference Material for KRAS Mutation Detection. Sci. Rep. 8, 9650. 10.1038/s41598-018-27368-3 30504843 PMC6269532

[B4] Findlay S. D. Vincent K. M. Berman J. R. Postovit L.-M. (2016). A Digital PCR-Based Method for Efficient and Highly Specific Screening of Genome Edited Cells. PLoS ONE 11 (4), e0153901. 10.1371/journal.pone.0153901 27089539 PMC4835065

[B5] Forghieri F. Nasillo V. Bettelli F. Pioli V. Giusti D. Gilioli A. (2020). Acute Myeloid Leukemia in Patients Living with HIV Infection: Several Questions, Fewer Answers. Ijms 21 (3), 1081. 10.3390/ijms21031081 PMC7036847 32041199

[B6] Gupta R. K. Peppa D. Hill A. L. Gálvez C. Salgado M. Pace M. (2020). Evidence for HIV-1 Cure after CCR5Δ32/Δ32 Allogeneic Haemopoietic Stem-Cell Transplantation 30 Months post Analytical Treatment Interruption: a Case Report. Lancet HIV 7 (5), E340–E347. 10.1016/S2352-3018(20)30069-2 32169158 PMC7606918

[B7] Henrich T. J. Gallien S. Li J. Z. Pereyra F. Kuritzkes D. R. (2012). Low-level Detection and Quantitation of Cellular HIV-1 DNA and 2-LTR Circles Using Droplet Digital PCR. J. Virol. Methods 186 (1-2), 68–72. 10.1016/j.jviromet.2012.08.019 22974526 PMC3517891

[B8] Hütter G. Neumann M. Nowak D. Klein S. Klüter H. Hofmann W.-K. (2011). The Effect of the CCR5-delta32 Deletion on Global Gene Expression Considering Immune Response and Inflammation. J. Inflamm. 8, 29. 10.1186/1476-9255-8-29 PMC3234179 22029606

[B9] Hütter G. Nowak D. Mossner M. Ganepola S. Müßig A. Allers K. (2009). Long-Term Control of HIV byCCR5Delta32/Delta32 Stem-Cell Transplantation. N. Engl. J. Med. 360 (7), 692–698. 10.1056/nejmoa0802905 19213682

[B10] Jilg N. Li J. Z. (2019). On the Road to a HIV Cure. Infect. Dis. Clin. North America 33 (3), 857–868. 10.1016/j.idc.2019.04.007 PMC6814144 31395147

[B11] Kageyama S. Mimaya J.-I. Yamada K. Kurimura T. Shiraki K. (2001). Polymorphism ofCCR5Affecting HIV Disease Progression in the Japanese Population. AIDS Res. Hum. Retroviruses 17 (11), 991–995. 10.1089/088922201300343663 11485615

[B12] Kang H. Minder P. Park M. A. Mesquitta W.-T. Torbett B. E. Slukvin II (2015). CCR5 Disruption in Induced Pluripotent Stem Cells Using CRISPR/Cas9 Provides Selective Resistance of Immune Cells to CCR5-Tropic HIV-1 Virus. Mol. Ther. - Nucleic Acids 4, e268. 10.1038/mtna.2015.42 26670276

[B13] Kang X. He W. Huang Y. Yu Q. Chen Y. Gao X. (2016). Introducing Precise Genetic Modifications into Human 3PN Embryos by CRISPR/Cas-mediated Genome Editing. J. Assist. Reprod. Genet. 33 (5), 581–588. 10.1007/s10815-016-0710-8 27052831 PMC4870449

[B14] Li H. Yang Y. Hong W. Huang M. Wu M. Zhao X. (2020). Applications of Genome Editing Technology in the Targeted Therapy of Human Diseases: Mechanisms, Advances and Prospects. Sig. Transduct. Target. Ther. 5 (1), 1. 10.1038/s41392-019-0089-y PMC6946647 32296011

[B15] Lin D. Scheller S. H. Robinson M. M. Izadpanah R. Alt E. U. Braun S. E. (2021). Increased Efficiency for Biallelic Mutations of the CCR5 Gene by CRISPR-Cas9 Using Multiple Guide RNAs as a Novel Therapeutic Option for Human Immunodeficiency Virus. CRISPR J. 4, 92–103. 10.1089/crispr.2020.0019 33616448 PMC8713505

[B16] Liu R. Paxton W. A. Choe S. Ceradini D. Martin S. R. Horuk R. (1996). Homozygous Defect in HIV-1 Coreceptor Accounts for Resistance of Some Multiply-Exposed Individuals to HIV-1 Infection. Cell 86 (3), 367–377. 10.1016/s0092-8674(00)80110-5 8756719

[B17] Liu Z. Chen S. Jin X. Wang Q. Yang K. Li C. (2017). Genome Editing of the HIV Co-receptors CCR5 and CXCR4 by CRISPR-Cas9 Protects CD4+ T Cells from HIV-1 Infection. Cell Biosci. 7, 47. 10.1186/s13578-017-0174-2 28904745 PMC5591563

[B18] Liu Z. Liang J. Chen S. Wang K. Liu X. Liu B. (2020). Genome Editing of CCR5 by AsCpf1 Renders CD4+T Cells Resistance to HIV-1 Infection. Cel. Biosci. 10, 85. 10.1186/s13578-020-00444-w PMC7346486 32670545

[B19] Malicherova B. Burjanivova T. Grendar M. Minarikova E. Bobrovska M. Vanova B. (2018). Droplet Digital PCR for Detection of BRAF V600E Mutation in Formalin-Fixed, Paraffin-Embedded Melanoma Tissues: a Comparison with Cobas® 4800, Sanger Sequencing, and Allele-specific PCR. Am. J. Transl. Res. 10 (11), 3773–3781. 30662627 PMC6291720

[B20] McEvoy A. C. Wood B. A. Ardakani N. M. Pereira M. R. Pearce R. Cowell L. (2018). Droplet Digital PCR for Mutation Detection in Formalin-Fixed, Paraffin-Embedded Melanoma Tissues. J. Mol. Diagn. 20 (2), 240–252. 10.1016/j.jmoldx.2017.11.009 29305225

[B21] Michael N. L. Chang G. Loum L. G. Mascola J. R. Dondero D. Birx D. L. (1997). The Role of Viral Phenotype and CCR-5 Gene Defects in HIV-1 Transmission and Disease Progression. Nat. Med. 3, 338–340. 10.1038/nm0397-338 9055864

[B22] Miyaoka Y. Berman J. R. Cooper S. B. Mayerl S. J. Chan A. H. Zhang B. (2016). Systematic Quantification of HDR and NHEJ Reveals Effects of Locus, Nuclease, and Cell Type on Genome-Editing. Sci. Rep., 6, 23549. 10.1038/srep23549 27030102 PMC4814844

[B23] Muxel S. M. Borelli S. D. Amarante M. K. Voltarelli J. C. Aoki M. N. de Oliveira C. E. C. C. E. (2008). Association Study of CCR5 delta 32 Polymorphism Among the HLA‐DRB1 Caucasian Population in Northern Paraná, Brazil. J. Clin. Lab. Anal. 22 (4), 229–233. 10.1002/jcla.20225 18623133 PMC6648960

[B24] Nischalke H. D. Nattermann J. Lichterfeld M. Woitas R. P. Rockstroh J. K. Sauerbruch T. (2004). Rapid Determination of the Δ32 Deletion in the Human CC-Chemokine Receptor 5 (CCR5) Gene without DNA Extraction by LightCycler Real-Time Polymerase Chain Reaction. AIDS Res. Hum. Retroviruses 20 (7), 750–754. 10.1089/0889222041524634 15307921

[B25] Qi C. Li D. Jiang X. Jia X. Lu L. Wang Y. (2018). Inducing CCR5Δ32/Δ32 Homozygotes in the Human Jurkat CD4+ Cell Line and Primary CD4+ Cells by CRISPR-Cas9 Genome-Editing Technology. Mol. Ther. - Nucleic Acids 12, 267–274. 10.1016/j.omtn.2018.05.012 30195765 PMC6005807

[B26] Quillent C. Oberlin E. Braun J. Rousset D. Gonzalez-Canali G. Métais P. (1998). HIV-1-resistance Phenotype Conferred by Combination of Two Separate Inherited Mutations of CCR5 Gene. The Lancet 351 (9095), 14–18. 10.1016/S0140-6736(97)09185-X 9433423

[B27] Reid A. L. Freeman J. B. Millward M. Ziman M. Gray E. S. (2015). Detection of BRAF-V600e and V600K in Melanoma Circulating Tumour Cells by Droplet Digital PCR. Clin. Biochem. 48, 1002. 10.1016/j.clinbiochem.2014.12.007 25523300

[B28] Ronsard L. Sood V. Yousif A. S. Ramesh J. Shankar V. Das J. (2019). Genetic Polymorphisms in the Open Reading Frame of the CCR5 Gene from HIV-1 Seronegative and Seropositive Individuals from National Capital Regions of India. Sci. Rep. 9, 7594. 10.1038/s41598-019-44136-z 31110236 PMC6527560

[B29] Rosi A. Meini G. Materazzi A. Vicenti I. Saladini F. Zazzi M. (2015). Low-cost Simultaneous Detection of CCR5-delta32 and HLA-B*5701 Alleles in Human Immunodeficiency Virus Type 1 Infected Patients by Selective Multiplex Endpoint PCR. J. Virol. Methods 224, 102–104. 10.1016/j.jviromet.2015.08.020 26341061

[B30] Schwarze L. I. Głów D. Sonntag T. Uhde A. Fehse B. (2021). Optimisation of a TALE Nuclease Targeting the HIV Co-receptor CCR5 for Clinical Application. Gene Ther. 28, 588–601. 10.1038/s41434-021-00271-9 34112993 PMC8455333

[B31] Stephens J. C. Reich D. E. Goldstein D. B. Shin H. D. Smith M. W. Carrington M. (1998). Dating the Origin of the CCR5-Δ32 AIDS-Resistance Allele by the Coalescence of Haplotypes. Am. J. Hum. Genet. 62, 1507–1515. 10.1086/301867 9585595 PMC1377146

[B32] Surdo M. Balestra E. Saccomandi P. Di Santo F. Montano M. Di Carlo D. (2013). Inhibition of Dual/Mixed Tropic HIV-1 Isolates by CCR5-Inhibitors in Primary Lymphocytes and Macrophages. PLoS ONE 8 (7), e68076. 10.1371/journal.pone.0068076 23874501 PMC3706609

[B33] Trecarichi E. M. Tumbarello M. Donati K. d. G. Tamburrini E. Cauda R. Brahe C. (2006). Partial Protective Effect of CCR5-Delta 32 Heterozygosity in a Cohort of Heterosexual Italian HIV-1 Exposed Uninfected Individuals. AIDS Res. Ther. 3, 22. 10.1186/1742-6405-3-22 16999868 PMC1592103

[B34] Venuti A. Pastori C. Lopalco L. (2017). The Role of Natural Antibodies to CC Chemokine Receptor 5 in HIV Infection. Front. Immunol. 8, 1358. 10.3389/fimmu.2017.01358 29163468 PMC5670346

[B35] Wang J. Zhao Y.-y. Li J.-f. Guo C.-c. Chen F.-r. Su H.-k. (2015). IDH1 Mutation Detection by Droplet Digital PCR in Glioma. Oncotarget 6 (637), 39651–39660. 10.18632/oncotarget.5630 26485760 PMC4741852

[B36] Watry H. L. Feliciano C. M. Gjoni K. Takahashi G. Miyaoka Y. Conklin B. R. (2020). Rapid, Precise Quantification of Large DNA Excisions and Inversions by ddPCR. Sci. Rep. 10, 14896. 10.1038/s41598-020-71742-z 32913194 PMC7483445

[B37] Whale A. S. Huggett J. F. Tzonev S. (2016). Fundamentals of Multiplexing with Digital PCR. Biomol. Detect. Quantification 10, 15–23. 10.1016/j.bdq.2016.05.002 PMC5154634 27990345

[B38] Xiao Q. Chen S. Wang Q. Liu Z. Liu S. Deng H. (2019). CCR5 Editing by *Staphylococcus aureus* Cas9 in Human Primary CD4+ T Cells and Hematopoietic Stem/progenitor Cells Promotes HIV-1 Resistance and CD4+ T Cell Enrichment in Humanized Mice. Retrovirology 16, 15. 10.1186/s12977-019-0477-y 31186067 PMC6560749

[B39] Ye J. Coulouris G. Zaretskaya I. Cutcutache I. Rozen S. Madden T. L. (2012). Primer-BLAST: A Tool to Design Target-specific Primers for Polymerase Chain Reaction. BMC Bioinformatics 13, 134. 10.1186/1471-2105-13-134 22708584 PMC3412702

[B40] Yu S. Yao Y. Xiao H. Li J. Liu Q. Yang Y. (2018). Simultaneous Knockout ofCXCR4andCCR5Genes in CD4+ T Cells via CRISPR/Cas9 Confers Resistance to Both X4- and R5-Tropic Human Immunodeficiency Virus Type 1 Infection. Hum. Gene Ther. 29, 51–67. 10.1089/hum.2017.032 28599597

[B41] Zheng B.-J. Zhao X.-Y. Zhu N.-S. Chan C.-P. Wong K.-H. Chan K. C.-W. (2002). Polymorphisms of CCR5 Gene in a Southern Chinese Population and Their Effects on Disease Progression in HIV Infections. AIDS 16 (18), 2480–2482. 10.1097/00002030-200212060-00016 12461425

