# Supplementary material for: Detection of CCR5Δ32 Mutant Alleles in Heterogeneous Cell Mixtures Using Droplet Digital PCR
Source: Front Mol Biosci. 2022 Feb 21;9:805931. doi: 10.3389/fmolb.2022.805931 (PMC8898955; doi:10.3389/fmolb.2022.805931)
Supplement: Supplementary file 2 [file DataSheet3.PDF]

Supplementary Materials

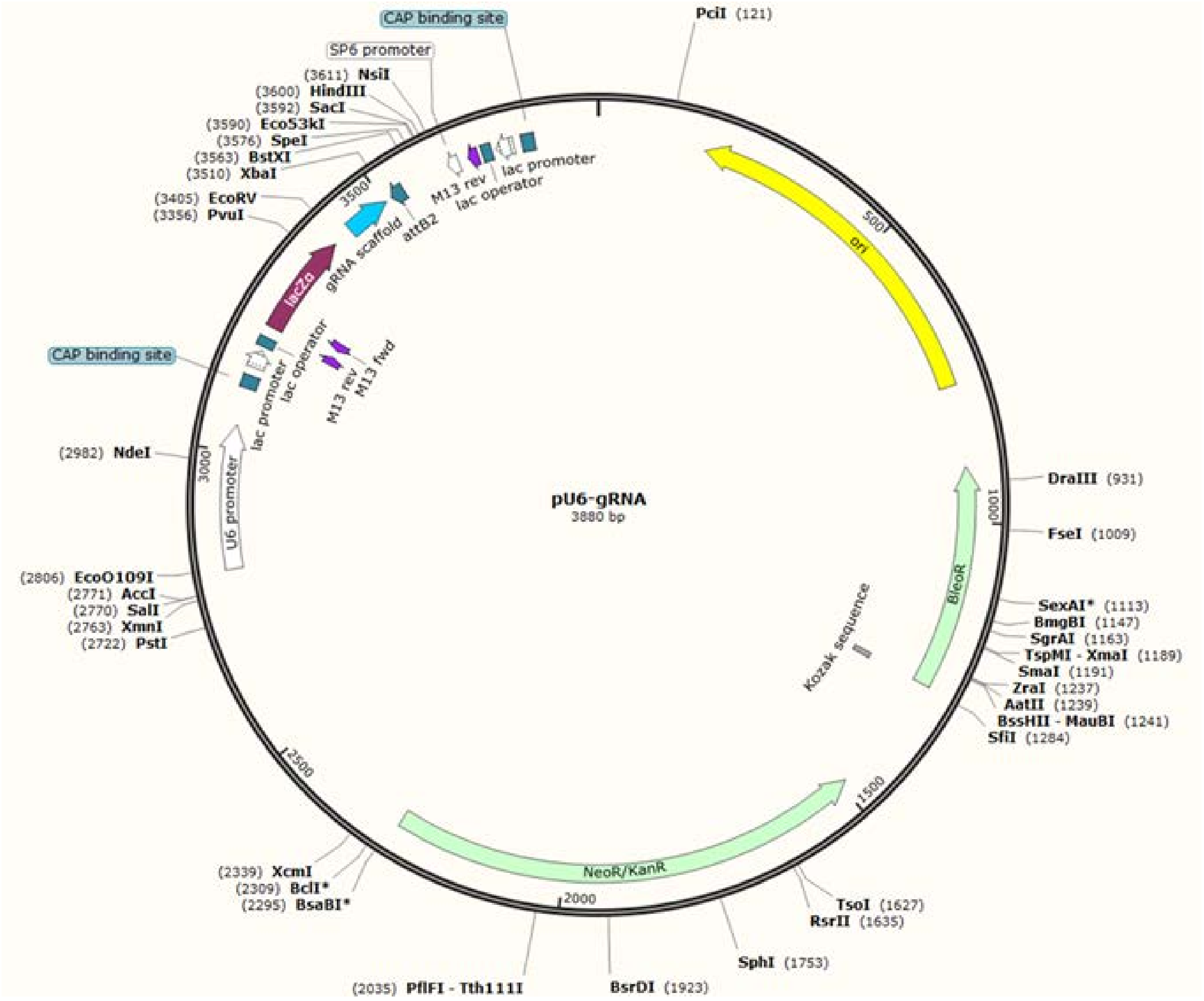

Suppl.Fig.1 pU6-gRNA circular map

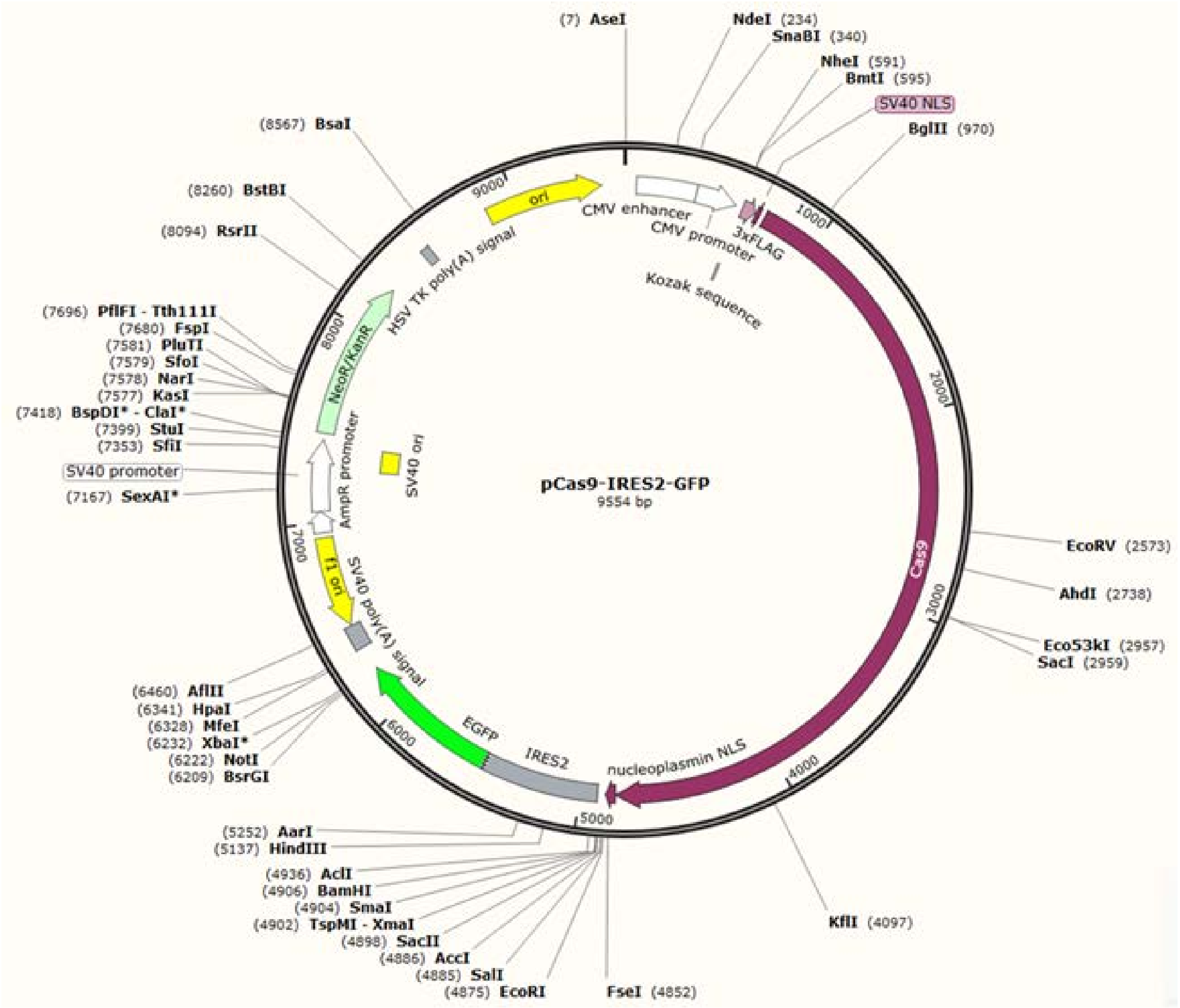

Suppl.Fig.2pCas9-IRES2-EGFP circular map

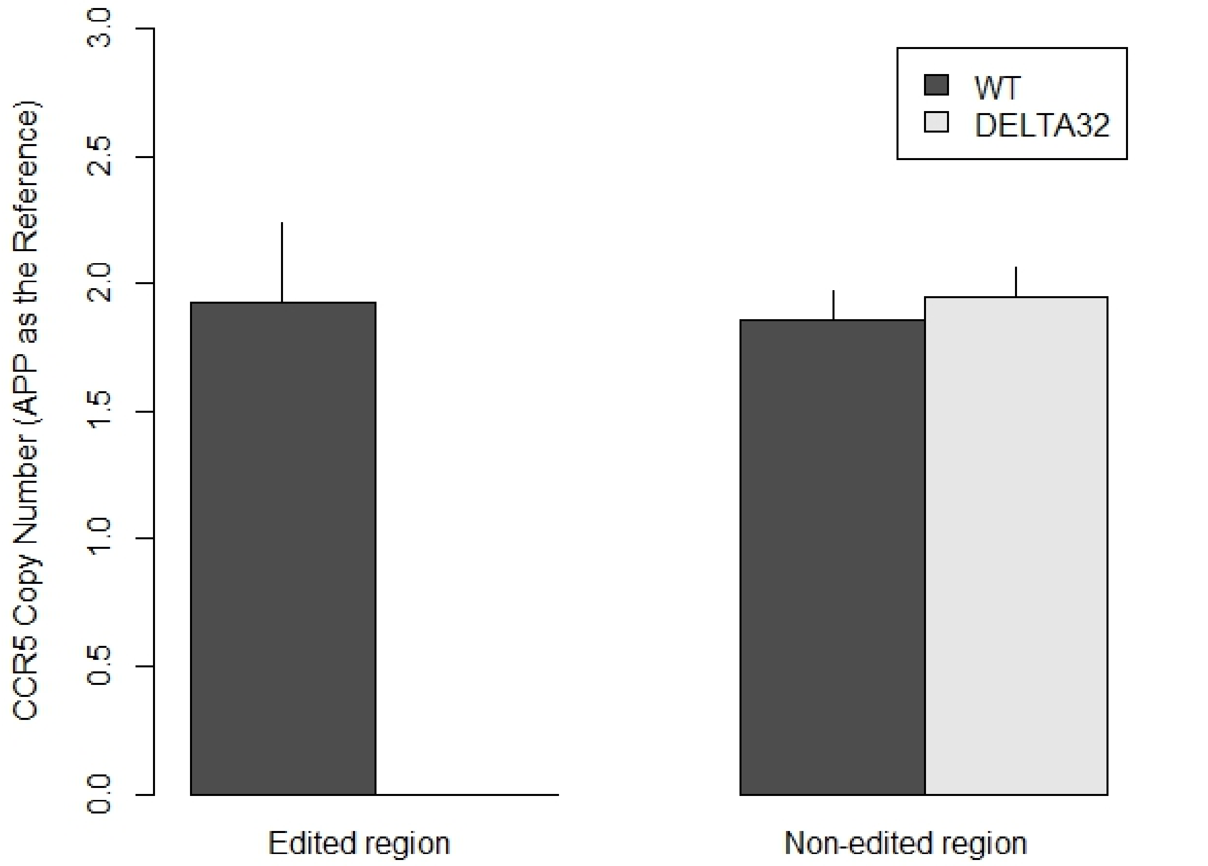

Suppl.Fig.3 Results of Copy Number Variation (CNV) assay by ddPCR in MT4-14 cell line.

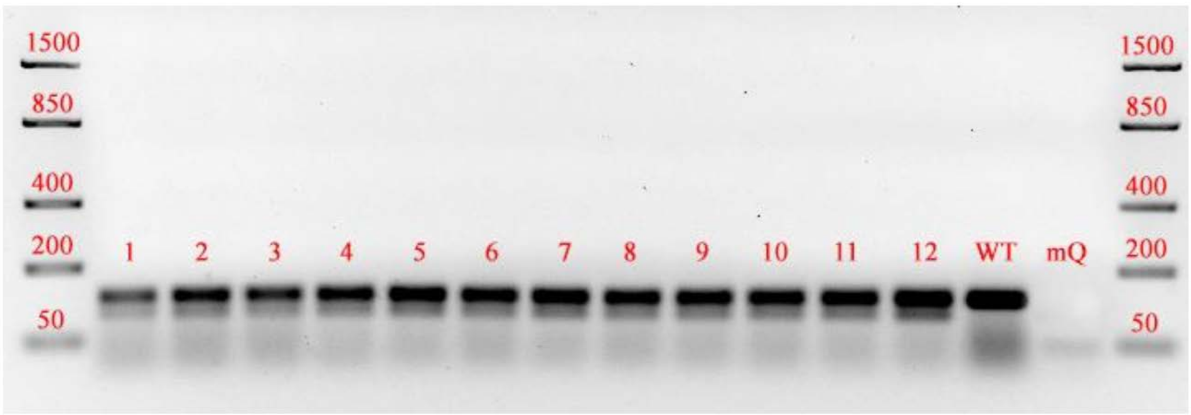

Suppl.Fig.4. PCR screening results of clones obtained by re-cloning the MT4-16 line with the putative heterozygous mutation CCR5delta32.

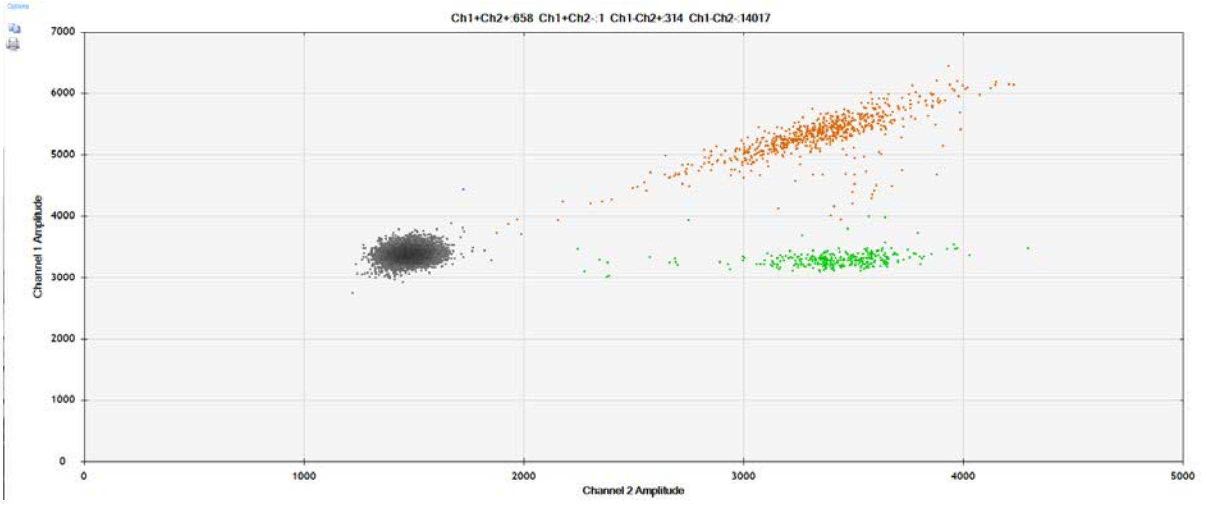

FAM (Mutant alleles), copies/ul	R6G (WT alleles), copies/ul	Ratio
139	255	0.545
142	273	0.520
161	161	0.537
160	160	0.537

Suppl.Fig.5. Results of ddPCR-assay on the MT4-16 line with a putative heterozygous CCR5delta32 mutation.

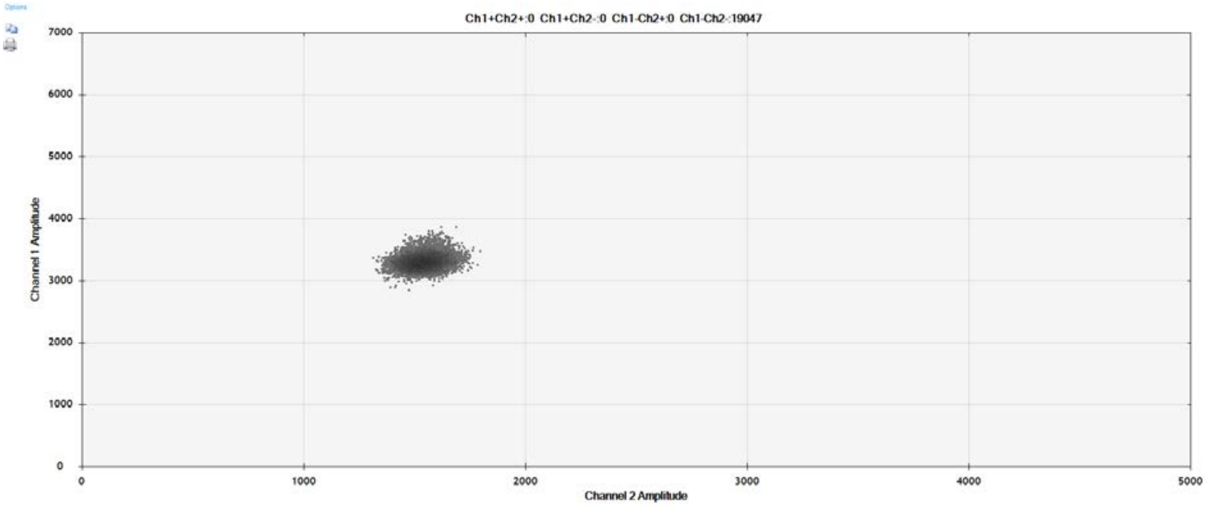

FAM (Mutant a lleles), copies/ul	R6G (WT alleles), copies/ul	Ratio
0	0	Not detected
0	0.17	0
0	0	Not detected
0	0	Not detected

Suppl.Fig.6. Results of ddPCR-assay on false-positive signals using NTC (no template control).

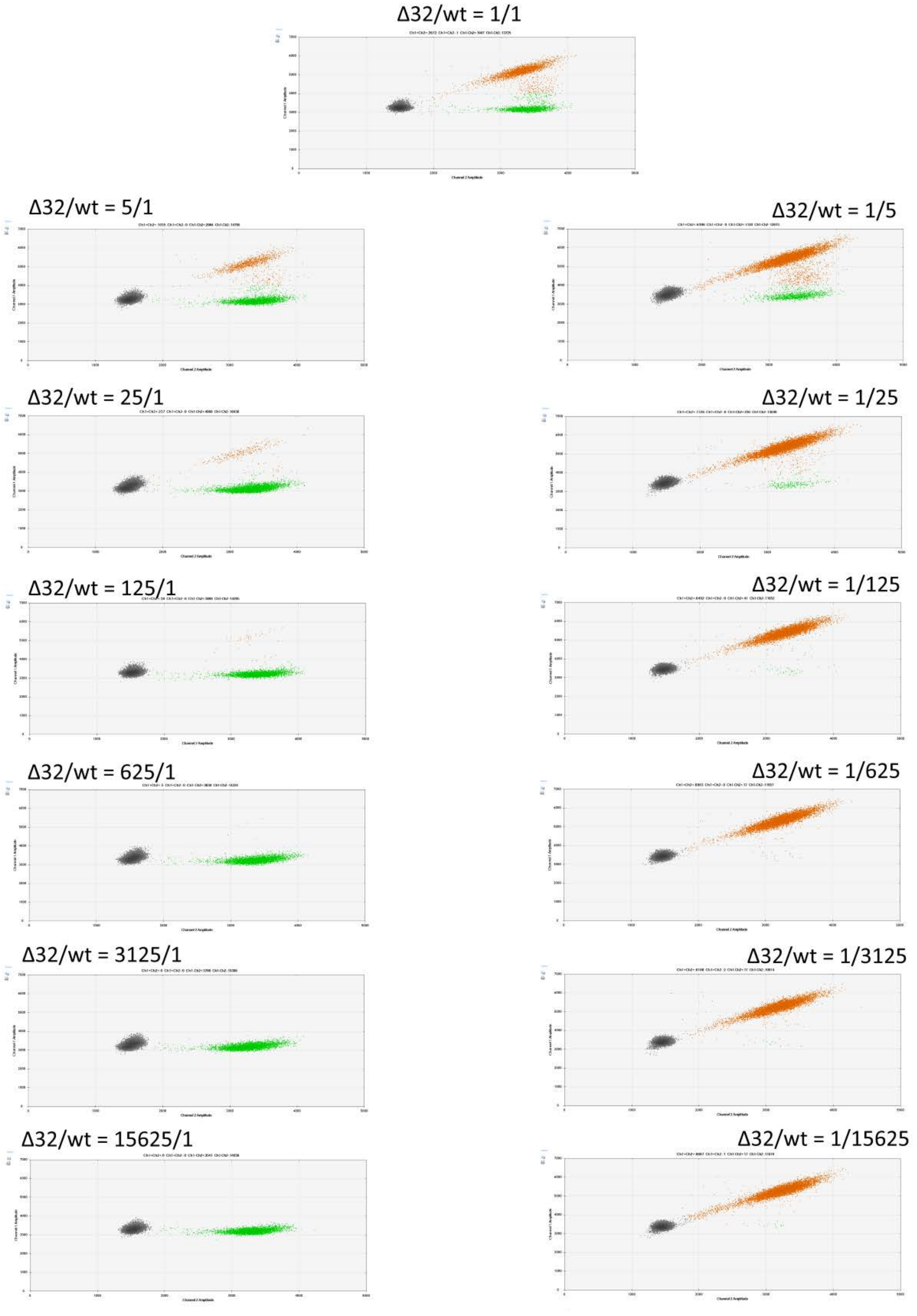

Suppl.Fig.7. 2-D visualization of ddPCR results of DNA dilution, DNA from Δ32 cells in wild-type DNA (the left-hand column) and wild-type DNA in DNA from Δ32 cells (the right-hand column).
