# Supplementary material for: Detection of CCR5Δ32 Mutant Alleles in Heterogeneous Cell Mixtures Using Droplet Digital PCR
Source: Front Mol Biosci. 2022 Feb 21;9:805931. doi: 10.3389/fmolb.2022.805931 (PMC8898955; doi:10.3389/fmolb.2022.805931)
Supplement: Supplementary file 3 [file DataSheet1.PDF]

pU6gRNA Cloning Vector 3519 bp DNA

```
   1 CTTCCGCTTC CTCGCTCACT GACTCGCTGC GCTCGGTCGT TCGGCTGCGG CGAGCGGTAT
  61 CAGCTCACTC AAAGGCGGTA ATACGGTTAT CCACAGAATC AGGGGATAAC GCAGGAAAGA
 121 ACATGTGAGC AAAAGGCCAG CAAAAGCCCA GGAACCGTAA AAAGGCCGCG TTGCTGGCGT
 181 TTTTCCATAG GCTCCGCCCC CCTGACGAGC ATCACAAAAA TCGACGCTCA AGTCAGAGGT
 241 GGCGAAACCC GACAGGACTA TAAAGATACC AGGCGTTTCC CCCTGGAAGC TCCCTCGTGC
 301 GCTCTCCTGT TCCGACCCTG CCGCTTACCG GATACCTGTC CGCCTTTCTC CCTTCGGGAA
 361 GCGTGGCGCT TTCTCATAGC TCACGCTGTA GGTATCTCAG TTCGGTGTAG GTCGTTCGCT
 421 CCAAGCTGGG CTGTGTGCAC GAACCCCCCG TTCAGCCCGA CCGCTGCGCC TTATCCGGTA
 481 ACTATCGTCT TGAGTCCAAC CCGGTAAGAC ACGACTTATC GCCACTGGCA GCAGCCACTG
 541 GTAACAGGAT TAGCAGAGCG AGGTATGTAG GCGGTGCTAC AGAGTTCTTG AAGTGGTGGC
 601 CTAACTACGG CTACACTAGA AGGACAGTAT TTGGTATCTG CGCTCTGCTG AAGCCAGTTA
 661 CCTTCGGAAA AAGAGTTGGT AGCTCTTGAT CCGGCAAACA AACCACCGCT GGTAGCGGTG
 721 GTTTTTTTGT TTGCAAGCAG CAGATTACGC GCAGAAAAAA AGGATCTCAA GAAGATCCTT
 781 TGATCTTTTC TACGGGGTCT GACGCTCAGT GGAACGAAAA CTCACGTTAA GGGATTTTGG
 841 TCATGAGATT ATCAAAAAGG ATCTTCACCT AGATCCTTTT AAATTAAAAA TGAAGTTTTA
 901 GCACGTGTCA GTCCTGCTCC TCGGCCACGA AGTGCACGCA GTTGCCGGCC GGGTCGCGCA
 961 GGGCGAACTC CCGCCCCCAC GGCTGCTCGC CGATCTCGGT CATGGCCGGC CCGGAGGCGT
1021 CCCGGAAGTT CGTGGACACG ACCTCCGACC ACTCGGCGTA CAGCTCGTCC AGGCCGCGCA
1081 CCCACACCCA GGCCAGGGTG TTGTCCGGCA CCACCTGGTC CTGGACCGCG CTGATGAACA
1141 GGGTCACGTC GTCCCGGACC ACACCGGCGA AGTCGTCCTC CACGAAGTCC CGGGAGAACC
1201 CGAGCCGGTC GGTCCAGAAC TCGACCGCTC CGGCGACGTC GCGCGCGGTG AGCACCGGAA
1261 CGGCACTGGT CAACTTGGCC ATGGTGGCCC TCCTCACGTG CTATTATTGA AGCATTTATC
1321 AGGGTTATTG TCTCATGAGC GGATACATAT TTGAATGTAT TTAGAAAAAT AAACAAATAG
1381 GGGTTCCGCG CACATTTCCC CGAAAAGTGC CACCTGTATG CGGTGTGAAA TACCGCACAG
1441 ATGCGTAAGG AGAAAATACC GCATCAGGAA ATTGTAAGCG TTAATAATTC AGAAGAACTC
1501 GTCAAGAAGG CGATAGAAGG CGATGCGCTG CGAATCGGGA GCGGCGATAC CGTAAAGCAC
1561 GAGGAAGCGG TCAGCCCATT CGCCGCCAAG CTCTTCAGCA ATATCACGGG TAGCCAACGC
1621 TATGTCCTGA TAGCGGTCCG CCACACCCAG CCGGCCACAG TCGATGAATC CAGAAAAGCG
1681 GCCATTTTCC ACCATGATAT TCGGCAAGCA GGCATCGCCA TGGGTCACGA CGAGATCCTC
1741 GCCGTCGGGC ATGCTCGCCT TGAGCCTGGC GAACAGTTCG GCTGGCGCGA GCCCCTGATG
1801 CTCTTCGTCC AGATCATCCT GATCGACAAG ACCGGCTTCC ATCCGAGTAC GTGCTCGCTC
1861 GATGCGATGT TTCGCTTGGT GGTCGAATGG GCAGGTAGCC GGATCAAGCG TATGCAGCCG
1921 CCGCATTGCA TCAGCCATGA TGGATACTTT CTCGGCAGGA GCAAGGTGAG ATGACAGGAG
1981 ATCCTGCCCC GGCACTTCGC CCAATAGCAG CCAGTCCCTT CCCGCTTCAG TGACAACGTC
2041 GAGCACAGCT GCGCAAGGAA CGCCCGTCGT GGCCAGCCAC GATAGCCGCG CTGCCTCGTC
2101 TTGCAGTTCA TTCAGGGCAC CGGACAGGTC GGTCTTGACA AAAAGAACCG GGCGCCCCTG
2161 CGCTGACAGC CGGAACACGG CGGCATCAGA GCAGCCGATT GTCTGTTGTG CCCAGTCATA
2221 GCCGAATAGC CTCTCCACCC AAGCGGCCGG AGAACCTGCG TGCAATCCAT CTTGTTCAAT
2281 CATGCGAAAC GATCCTCATC CTGTCTCTTG ATCAGAGCTT GATCCCCTGC GCCATCAGAT
2341 CCTTGGCGGC GAGAAAGCCA TCCAGTTTAC TTTGCAGGGC TTCCCAACCT TACCAGAGGG
2401 CGCCCCAGCT GGCAATTCCG GTTCGCTTGC TGTCCATAAA ACCGCCCAGT CTAGCTATCG
2461 CCATGTAAGC CCACTGCAAG CTACCTGCTT TCTCTTTGCG CTTGCGTTTT CCCTTGTCCA
2521 GATAGCCCAG TAGCTGACAT TCATCCGGGG TCAGCACCGT TTCTGCGGAC TGGCTTTCTA
2581 CGTGAAAAGG ATCTAGGTGA AGATCCTTTT TGATAATCTC ATGCCTGACA TTTATATTCC
2641 CCAGAACATC AGGTTAATGG CGTTTTTGAT GTCATTTTCG CGGTGGCTGA GATCAGCCAC
2701 TTCTTCCCCG ATAACATCTG CAGAATTCGC CCTTTGTACA AAAAAGCAGG CTTTAAAGGA
2761 ACCAATTCAG TCGACTGGAT CCGGTACCAA GGTCGGGCAG GAAGAGGGCC TATTTCCCAT
2821 GATTCCTTCA TATTTGCATA TACGATACAA GGCTGTTAGA GAGATAATTA GAATTAATTT
2881 GACTGTAAAC ACAAAGATAT TAGTACAAAA TACGTGACGT AGAAAGTAAT AATTTCTTGG
2941 GTAGTTTGCA GTTTTAAAAT TATGTTTTAA AATGGACTAT CATATGCTTA CCGTAACTTG
3001 AAAGTATTTC gatttcttgg ctttatatat cttGTGGAAA GGACGAAACA CCggGTCTTC
3061 gcacgacagg tttcccgact ggaaagcggg cagtgagcgc aacgcaatta atgtgagtta
3121 gctcactcat taggcacccc aggctttaca ctttatgctt ccggctcgta tgttgtgtgg
3181 aattgtgagc ggataacaat ttcacacagg aaacagctat gaccatgatt acggattcac
3241 tggccgtcgt tttacaacgt cgtgactggg aaaaccctgg cgttacccaa cttaatcgcc
3301 ttgcagcaca tccccctttc gccagctggc gtaatagcga agaggcccgc accgatcgcc
3361 cttcccaaca gttgcgcagc ctgaatggcg aatggcgcgA ACgatatcGT GCggatccaG
3421 AAGACctgtt ttagagctaG AAAtagcaag ttaaaataag gctagtcCGT TATCAACTTG
```

```
3481 AAAAAGTGGC ACCGAGTCGG TGCTTTTTTT CTAGACCCAG CTTTCTTGTA CAAAGTTGGC
3541 ATTAAAGGGC GAATTCCAGC ACACTGGCGG CCGTTACTAG TGGATCCGAG CTCGGTACCA
3601 AGCTTGATGC ATAGCTTGAG TATTCTATAG TGTCACCTAA ATAGCTTGGC GTAATCATGG
3661 TCATAGCTGT TTCCTGTGTG AAATTGTTAT CCGCTCACAA TTCCACACAA CATACGAGCC
3721 GGAAGCATAA AGTGTAAAGC CTGGGGTGCC TAATGAGTGA GCTAACTCAC ATTAATTGCG
3781 TTGCGCTCAC TGCCCGCTTT CCAGTCGGGA AACCTGTCGT GCCAGCTGCA TTAATGAATC
3841 GGCCAACGCG CGGGGAGAGG CGGTTTGCGT ATTGGGCGCT
//
```
